# 3,6-Dibromo­naphthalene-2,7-diyl bis­(trifluoro­methane­sulfonate)

**DOI:** 10.1107/S1600536811037755

**Published:** 2011-09-30

**Authors:** Xiang-Xiang Wu, Yan Wan, Seik Weng Ng

**Affiliations:** a Henan University of Traditional Chinese Medicine, Zhengzhou 450008, People’s Republic of China; b Department of Chemistry, University of Malaya, 50603 Kuala Lumpur, Malaysia; c Chemistry Department, Faculty of Science, King Abdulaziz University, PO Box 80203 Jeddah, Saudi Arabia

## Abstract

The naphthalene fused ring of the title compound, C_12_H_4_Br_2_F_6_O_6_S_2_, is slightly buckled (r.m.s. deviation = 0.036 Å) along the common C—C bond and the benzene rings are twisted by 3.2 (3)°. The two trifluoro­methyl­sulfonyl groups lie on opposite sides of the fused-ring system. The crystal structure features short inter­molecular F⋯F contacts [2.715 (4) and 2.832 (4) Å].

## Related literature

For the synthesis and background chemistry, see: Shinamura *et al.* (2011 ▶).
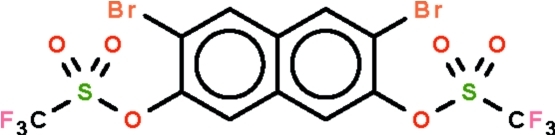


## Experimental

### 

#### Crystal data

C_12_H_4_Br_2_F_6_O_6_S_2_
*M* *_r_* = 582.09
Monoclinic, 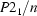

*a* = 5.2413 (11) Å
*b* = 26.450 (6) Å
*c* = 12.429 (3) Å
β = 90.169 (3)°
*V* = 1723.1 (7) Å^3^
*Z* = 4
Mo *K*α radiation
μ = 5.04 mm^−1^
*T* = 173 K
0.32 × 0.30 × 0.20 mm

#### Data collection

Rigaku Saturn724+ CCD diffractometer
Absorption correction: multi-scan (*CrystalClear*; Rigaku, 2007 ▶) *T*_min_ = 0.573, *T*_max_ = 1.000
17398 measured reflections
3933 independent reflections
3662 reflections with *I* > 2σ(*I*)
*R*_int_ = 0.048

#### Refinement

*R*[*F* ^2^ > 2σ(*F* ^2^)] = 0.044
*wR*(*F* ^2^) = 0.094
*S* = 1.16
3933 reflections
253 parameters
H-atom parameters constrained
Δρ_max_ = 0.79 e Å^−3^
Δρ_min_ = −0.45 e Å^−3^

### 

Data collection: *CrystalClear* (Rigaku, 2007 ▶); cell refinement: *CrystalClear*; data reduction: *CrystalClear*; program(s) used to solve structure: *SHELXS97* (Sheldrick, 2008 ▶); program(s) used to refine structure: *SHELXL97* (Sheldrick, 2008 ▶); molecular graphics: *X-SEED* (Barbour, 2001 ▶); software used to prepare material for publication: *publCIF* (Westrip, 2010 ▶).

## Supplementary Material

Crystal structure: contains datablock(s) global, I. DOI: 10.1107/S1600536811037755/nk2112sup1.cif

Structure factors: contains datablock(s) I. DOI: 10.1107/S1600536811037755/nk2112Isup2.hkl

Supplementary material file. DOI: 10.1107/S1600536811037755/nk2112Isup3.cml

Additional supplementary materials:  crystallographic information; 3D view; checkCIF report

## Figures and Tables

**Fig. 1.**
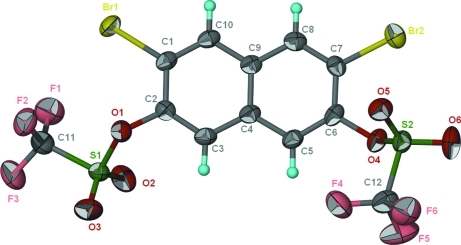
Displacement ellipsoid plot (Barbour, 2001) of C12H4Br2F6O6S2 at the 70% probability level; hydrogen atoms are drawn as spheres of arbitrary radius.

## supplementary crystallographic information

### Comment

The title compound (Scheme I), which is synthesized from commercially available 3,6-dibromonaphthalene-2,7-diol, has trifluoromethysulfonyloxy (abbreviated OTf) substituents that can be replaced by an acetylenic –C≡C–*R* radical. The product is then converted to a naphthodithiophene by treatment with sodium sulfide. Such naphthodithiophenes can be used as an organic field-effect transistors (Shinamura *et al.*, 2011). We intend to examine these compounds for such applications; the title compound is a precursor. The naphthalene fused-ring is slightly buckled along the common carbon-carbon bond as the benzene rings are offset by 3.2 (3) °. The two trifluoromethylsulfonyl groups lie on opposite sides of the fused-ring (Fig. 1). The crystal structure features short intermolecular F···F contacts (2.715 (4) Å between F3 and inversion-related F4, and 2.832 (4) Å between F3 and inversion symmetry-related F5) that give rise to the formation of a ribbon motif.

### Experimental

3,6-Dibromonaphthalene-2,7-diol (3.15 g,10 mmol) was dissolved in the mixture of dichloromethane (100 ml) and triethylamine (3.04 g,30 mmol). The solution was cooled to 273 K. Trifluoromethanesulfonic acid anhydride (3.21 g, 22 mmol) dissolved in dichloromethane (20 ml) was added dropwise. The mxiture was kept cold for 12 h. The solvent was evaporated and the residue purified by column chromatography on silica gel (petroleum ether:ethyl acetate =10:1) to provide the desired product as a white solid (3.78 g, yield 65%). The procedure was that reported by Shinamura *et al.* (2011).

### Refinement

Carbon-bound H-atoms were placed in calculated positions [C—H 0.95 Å, *U*_iso_(H) 1.2*U*_eq_(C)] and were included in the refinement in the riding model approximation.  Omitted from the refinement because of bad disagreement between observed and calculated structure factors were reflections (0 1 1), (0 2 0) and (0 2 1).

### Crystal data

**Table:**

C_12_H_4_Br_2_F_6_O_6_S_2_	*F*(000) = 1120
*M**_r_* = 582.09	*D*_x_ = 2.244 Mg m^−^^3^
Monoclinic, *P*2_1_/*n*	Mo *K*α radiation, λ = 0.71073 Å
Hall symbol: -P 2yn	Cell parameters from 5210 reflections
*a* = 5.2413 (11) Å	θ = 0.8–27.5°
*b* = 26.450 (6) Å	µ = 5.04 mm^−^^1^
*c* = 12.429 (3) Å	*T* = 173 K
β = 90.169 (3)°	Prism, colourless
*V* = 1723.1 (7) Å^3^	0.32 × 0.30 × 0.20 mm
*Z* = 4	

### Data collection

**Table:**

Rigaku Saturn724+ CCD diffractometer	3933 independent reflections
Radiation source: fine-focus sealed tube	3662 reflections with *I* > 2σ(*I*)
graphite	*R*_int_ = 0.048
ω scans at fixed χ = 45°	θ_max_ = 27.5°, θ_min_ = 2.8°
Absorption correction: multi-scan (*CrystalClear*; Rigaku, 2007)	*h* = −6→6
*T*_min_ = 0.573, *T*_max_ = 1.000	*k* = −34→34
17398 measured reflections	*l* = −16→16

### Refinement

**Table:**

Refinement on *F*^2^	Primary atom site location: structure-invariant direct methods
Least-squares matrix: full	Secondary atom site location: difference Fourier map
*R*[*F*^2^ > 2σ(*F*^2^)] = 0.044	Hydrogen site location: inferred from neighbouring sites
*wR*(*F*^2^) = 0.094	H-atom parameters constrained
*S* = 1.16	*w* = 1/[σ^2^(*F*_o_^2^) + (0.0349*P*)^2^ + 2.4738*P*] where *P* = (*F*_o_^2^ + 2*F*_c_^2^)/3
3933 reflections	(Δ/σ)_max_ = 0.001
253 parameters	Δρ_max_ = 0.79 e Å^−^^3^
0 restraints	Δρ_min_ = −0.45 e Å^−^^3^

### Special details

**Table:**

Geometry. All e.s.d.'s (except the e.s.d. in the dihedral angle between two l.s. planes) are estimated using the full covariance matrix. The cell e.s.d.'s are taken into account individually in the estimation of e.s.d.'s in distances, angles and torsion angles; correlations between e.s.d.'s in cell parameters are only used when they are defined by crystal symmetry. An approximate (isotropic) treatment of cell e.s.d.'s is used for estimating e.s.d.'s involving l.s. planes.

### Fractional atomic coordinates and isotropic or equivalent isotropic displacement parameters (Å^2^)

**Table:**

	*x*	*y*	*z*	*U*_iso_*/*U*_eq_	
Br1	−0.38667 (8)	0.159089 (15)	0.15955 (3)	0.03395 (12)	
Br2	0.69892 (7)	0.232466 (15)	0.59710 (3)	0.03228 (12)	
S1	−0.51801 (18)	0.01955 (4)	0.30367 (8)	0.0297 (2)	
S2	0.36288 (18)	0.17543 (4)	0.81831 (7)	0.0286 (2)	
F1	−0.4753 (6)	0.03529 (11)	0.0989 (2)	0.0519 (7)	
F2	−0.8431 (5)	0.05488 (10)	0.1624 (2)	0.0486 (7)	
F3	−0.7393 (6)	−0.02313 (10)	0.1425 (2)	0.0576 (8)	
F4	−0.0166 (5)	0.11242 (10)	0.8258 (2)	0.0501 (7)	
F5	0.3216 (6)	0.08682 (11)	0.9073 (2)	0.0634 (8)	
F6	0.0909 (5)	0.14491 (11)	0.9781 (2)	0.0506 (7)	
O1	−0.5105 (5)	0.07808 (10)	0.3257 (2)	0.0295 (6)	
O2	−0.2705 (6)	−0.00101 (11)	0.2966 (3)	0.0435 (7)	
O3	−0.7065 (6)	−0.00149 (13)	0.3702 (3)	0.0523 (9)	
O4	0.4608 (5)	0.14368 (10)	0.71859 (19)	0.0296 (6)	
O5	0.1917 (5)	0.21376 (10)	0.7854 (2)	0.0339 (6)	
O6	0.5748 (6)	0.18540 (13)	0.8852 (2)	0.0453 (8)	
C1	−0.2103 (7)	0.14519 (14)	0.2887 (3)	0.0246 (7)	
C2	−0.2856 (7)	0.10442 (14)	0.3545 (3)	0.0243 (7)	
C3	−0.1645 (7)	0.09387 (13)	0.4487 (3)	0.0260 (7)	
H3	−0.2168	0.0658	0.4910	0.031*	
C4	0.0403 (7)	0.12493 (13)	0.4835 (3)	0.0247 (7)	
C5	0.1600 (7)	0.11762 (14)	0.5845 (3)	0.0264 (8)	
H5	0.1130	0.0900	0.6292	0.032*	
C6	0.3434 (7)	0.15073 (14)	0.6165 (3)	0.0260 (8)	
C7	0.4289 (7)	0.19063 (13)	0.5502 (3)	0.0247 (7)	
C8	0.3173 (7)	0.19787 (14)	0.4520 (3)	0.0263 (7)	
H8	0.3742	0.2245	0.4068	0.032*	
C9	0.1171 (7)	0.16576 (13)	0.4172 (3)	0.0239 (7)	
C10	−0.0091 (7)	0.17479 (14)	0.3192 (3)	0.0249 (7)	
H10	0.0457	0.2016	0.2740	0.030*	
C11	−0.6552 (9)	0.02223 (16)	0.1672 (3)	0.0374 (9)	
C12	0.1767 (9)	0.12624 (16)	0.8865 (3)	0.0376 (9)	

### Atomic displacement parameters (Å^2^)

**Table:**

	*U*^11^	*U*^22^	*U*^33^	*U*^12^	*U*^13^	*U*^23^
Br1	0.0434 (2)	0.0326 (2)	0.0258 (2)	0.00171 (17)	−0.00934 (16)	0.00457 (15)
Br2	0.0305 (2)	0.0321 (2)	0.0343 (2)	−0.00321 (15)	0.00105 (15)	−0.00809 (15)
S1	0.0307 (5)	0.0236 (5)	0.0348 (5)	−0.0030 (4)	−0.0049 (4)	0.0053 (4)
S2	0.0322 (5)	0.0296 (5)	0.0239 (4)	0.0006 (4)	−0.0044 (4)	−0.0032 (4)
F1	0.0710 (19)	0.0505 (17)	0.0342 (14)	−0.0086 (14)	0.0094 (13)	−0.0054 (12)
F2	0.0517 (16)	0.0439 (15)	0.0501 (15)	0.0067 (12)	−0.0195 (12)	0.0014 (12)
F3	0.072 (2)	0.0351 (15)	0.0652 (18)	−0.0132 (14)	−0.0185 (15)	−0.0114 (13)
F4	0.0533 (16)	0.0500 (17)	0.0472 (15)	−0.0194 (13)	0.0033 (12)	−0.0053 (12)
F5	0.083 (2)	0.0451 (17)	0.0623 (18)	0.0213 (15)	0.0097 (16)	0.0221 (14)
F6	0.0611 (17)	0.0595 (18)	0.0313 (13)	−0.0012 (14)	0.0112 (12)	−0.0017 (12)
O1	0.0299 (13)	0.0263 (14)	0.0321 (14)	0.0015 (11)	−0.0038 (11)	−0.0013 (11)
O2	0.0354 (16)	0.0268 (15)	0.068 (2)	0.0047 (12)	−0.0101 (14)	−0.0043 (14)
O3	0.0495 (19)	0.056 (2)	0.0515 (19)	−0.0178 (16)	−0.0005 (15)	0.0233 (16)
O4	0.0347 (14)	0.0324 (15)	0.0218 (12)	0.0063 (11)	−0.0046 (10)	−0.0033 (10)
O5	0.0431 (16)	0.0275 (14)	0.0312 (14)	0.0057 (12)	−0.0002 (12)	−0.0021 (11)
O6	0.0403 (16)	0.059 (2)	0.0362 (16)	0.0000 (15)	−0.0135 (13)	−0.0151 (14)
C1	0.0309 (19)	0.0239 (18)	0.0191 (16)	0.0057 (15)	0.0018 (14)	−0.0001 (13)
C2	0.0241 (17)	0.0225 (18)	0.0263 (18)	0.0016 (14)	0.0020 (14)	−0.0025 (14)
C3	0.036 (2)	0.0194 (17)	0.0228 (17)	0.0014 (15)	0.0035 (14)	0.0012 (13)
C4	0.0329 (19)	0.0198 (17)	0.0213 (17)	0.0040 (15)	0.0016 (14)	−0.0008 (13)
C5	0.036 (2)	0.0210 (18)	0.0219 (17)	0.0040 (15)	−0.0018 (14)	0.0002 (14)
C6	0.0307 (19)	0.030 (2)	0.0176 (16)	0.0070 (15)	−0.0020 (14)	−0.0015 (14)
C7	0.0259 (17)	0.0194 (17)	0.0289 (18)	0.0007 (14)	−0.0002 (14)	−0.0055 (14)
C8	0.0316 (19)	0.0209 (18)	0.0265 (18)	0.0026 (15)	0.0050 (14)	−0.0013 (14)
C9	0.0311 (19)	0.0171 (17)	0.0237 (17)	0.0053 (14)	0.0026 (14)	−0.0026 (13)
C10	0.0321 (19)	0.0227 (18)	0.0200 (16)	0.0053 (15)	0.0042 (14)	0.0018 (13)
C11	0.044 (2)	0.028 (2)	0.040 (2)	−0.0033 (18)	−0.0039 (19)	−0.0027 (17)
C12	0.048 (2)	0.034 (2)	0.031 (2)	0.0055 (19)	0.0030 (18)	0.0018 (17)

### Geometric parameters (Å, °)

**Table:**

Br1—C1	1.886 (3)	O4—C6	1.421 (4)
Br2—C7	1.887 (3)	C1—C10	1.367 (5)
S1—O3	1.405 (3)	C1—C2	1.410 (5)
S1—O2	1.409 (3)	C2—C3	1.359 (5)
S1—O1	1.573 (3)	C3—C4	1.418 (5)
S1—C11	1.841 (4)	C3—H3	0.9500
S2—O6	1.410 (3)	C4—C5	1.415 (5)
S2—O5	1.414 (3)	C4—C9	1.417 (5)
S2—O4	1.584 (3)	C5—C6	1.359 (5)
S2—C12	1.835 (5)	C5—H5	0.9500
F1—C11	1.317 (5)	C6—C7	1.412 (5)
F2—C11	1.311 (5)	C7—C8	1.366 (5)
F3—C11	1.314 (5)	C8—C9	1.417 (5)
F4—C12	1.313 (5)	C8—H8	0.9500
F5—C12	1.315 (5)	C9—C10	1.405 (5)
F6—C12	1.321 (5)	C10—H10	0.9500
O1—C2	1.414 (4)		
			
O3—S1—O2	122.2 (2)	C4—C5—H5	120.6
O3—S1—O1	107.7 (2)	C5—C6—C7	122.4 (3)
O2—S1—O1	111.60 (16)	C5—C6—O4	118.7 (3)
O3—S1—C11	106.5 (2)	C7—C6—O4	118.8 (3)
O2—S1—C11	108.3 (2)	C8—C7—C6	119.3 (3)
O1—S1—C11	97.59 (16)	C8—C7—Br2	120.8 (3)
O6—S2—O5	122.32 (19)	C6—C7—Br2	119.8 (3)
O6—S2—O4	107.69 (17)	C7—C8—C9	120.2 (3)
O5—S2—O4	111.13 (15)	C7—C8—H8	119.9
O6—S2—C12	106.2 (2)	C9—C8—H8	119.9
O5—S2—C12	107.73 (19)	C10—C9—C8	120.6 (3)
O4—S2—C12	99.18 (17)	C10—C9—C4	120.0 (3)
C2—O1—S1	123.3 (2)	C8—C9—C4	119.4 (3)
C6—O4—S2	119.3 (2)	C1—C10—C9	120.3 (3)
C10—C1—C2	119.6 (3)	C1—C10—H10	119.8
C10—C1—Br1	120.1 (3)	C9—C10—H10	119.8
C2—C1—Br1	120.4 (3)	F3—C11—F2	109.8 (4)
C3—C2—C1	121.7 (3)	F3—C11—F1	109.2 (4)
C3—C2—O1	120.2 (3)	F2—C11—F1	109.7 (4)
C1—C2—O1	117.7 (3)	F3—C11—S1	108.1 (3)
C2—C3—C4	119.6 (3)	F2—C11—S1	111.0 (3)
C2—C3—H3	120.2	F1—C11—S1	109.0 (3)
C4—C3—H3	120.2	F4—C12—F5	109.7 (4)
C5—C4—C9	119.6 (3)	F4—C12—F6	109.5 (4)
C5—C4—C3	121.6 (3)	F5—C12—F6	108.9 (3)
C9—C4—C3	118.7 (3)	F4—C12—S2	110.0 (3)
C6—C5—C4	118.9 (3)	F5—C12—S2	110.2 (3)
C6—C5—H5	120.6	F6—C12—S2	108.4 (3)
			
O3—S1—O1—C2	125.8 (3)	C7—C8—C9—C10	176.1 (3)
O2—S1—O1—C2	−10.9 (3)	C7—C8—C9—C4	−2.4 (5)
C11—S1—O1—C2	−124.1 (3)	C5—C4—C9—C10	−176.9 (3)
O6—S2—O4—C6	−146.3 (3)	C3—C4—C9—C10	0.0 (5)
O5—S2—O4—C6	−9.9 (3)	C5—C4—C9—C8	1.7 (5)
C12—S2—O4—C6	103.3 (3)	C3—C4—C9—C8	178.5 (3)
C10—C1—C2—C3	0.6 (6)	C2—C1—C10—C9	−2.1 (5)
Br1—C1—C2—C3	−179.1 (3)	Br1—C1—C10—C9	177.7 (3)
C10—C1—C2—O1	173.5 (3)	C8—C9—C10—C1	−176.8 (3)
Br1—C1—C2—O1	−6.2 (4)	C4—C9—C10—C1	1.8 (5)
S1—O1—C2—C3	−63.9 (4)	O3—S1—C11—F3	−53.1 (4)
S1—O1—C2—C1	123.0 (3)	O2—S1—C11—F3	80.0 (3)
C1—C2—C3—C4	1.1 (5)	O1—S1—C11—F3	−164.2 (3)
O1—C2—C3—C4	−171.6 (3)	O3—S1—C11—F2	67.4 (4)
C2—C3—C4—C5	175.4 (3)	O2—S1—C11—F2	−159.5 (3)
C2—C3—C4—C9	−1.4 (5)	O1—S1—C11—F2	−43.7 (3)
C9—C4—C5—C6	1.2 (5)	O3—S1—C11—F1	−171.6 (3)
C3—C4—C5—C6	−175.5 (3)	O2—S1—C11—F1	−38.5 (3)
C4—C5—C6—C7	−3.5 (5)	O1—S1—C11—F1	77.3 (3)
C4—C5—C6—O4	179.2 (3)	O6—S2—C12—F4	−176.0 (3)
S2—O4—C6—C5	−97.9 (4)	O5—S2—C12—F4	51.3 (3)
S2—O4—C6—C7	84.7 (4)	O4—S2—C12—F4	−64.5 (3)
C5—C6—C7—C8	2.8 (6)	O6—S2—C12—F5	−55.0 (3)
O4—C6—C7—C8	−179.9 (3)	O5—S2—C12—F5	172.4 (3)
C5—C6—C7—Br2	−176.1 (3)	O4—S2—C12—F5	56.6 (3)
O4—C6—C7—Br2	1.2 (4)	O6—S2—C12—F6	64.2 (3)
C6—C7—C8—C9	0.3 (5)	O5—S2—C12—F6	−68.5 (3)
Br2—C7—C8—C9	179.2 (3)	O4—S2—C12—F6	175.8 (3)
